# Supplementary material for: DNA methylation repels binding of hypoxia-inducible transcription factors to maintain tumor immunotolerance
Source: Genome Biol. 2020 Jul 27;21:182. doi: 10.1186/s13059-020-02087-z (PMC7384226; doi:10.1186/s13059-020-02087-z)
Supplement: Supplementary file 1 — Additional file 1: Fig. S1. HIF1β peaks in MCF7 cells under 0.5% O2. Fig. S2. Expression of hypoxia genes and cancer testis antigens. Fig. S3. Cell-type-specific HIF1β binding. Fig. S4. DNA methylation directly repels HIF1β binding. Fig. S5. Quality control of the ChIP-seq replicates. Fig. S6. HIF binds retrotransposons in demethylated genomes. Fig. S7. Examples of cryptic transcripts upregulated by HIF1β. Fig. S8. Cryptic transcript expression in TCGA tumors. Fig. S9. Aza treatment increases immunogenicity. [file 13059_2020_2087_MOESM1_ESM.pdf]

SUPPLEMENTARY FIGURES

DNA methylation repels binding of hypoxia-inducible transcription factors to maintain tumour immunotolerance

Figure S1

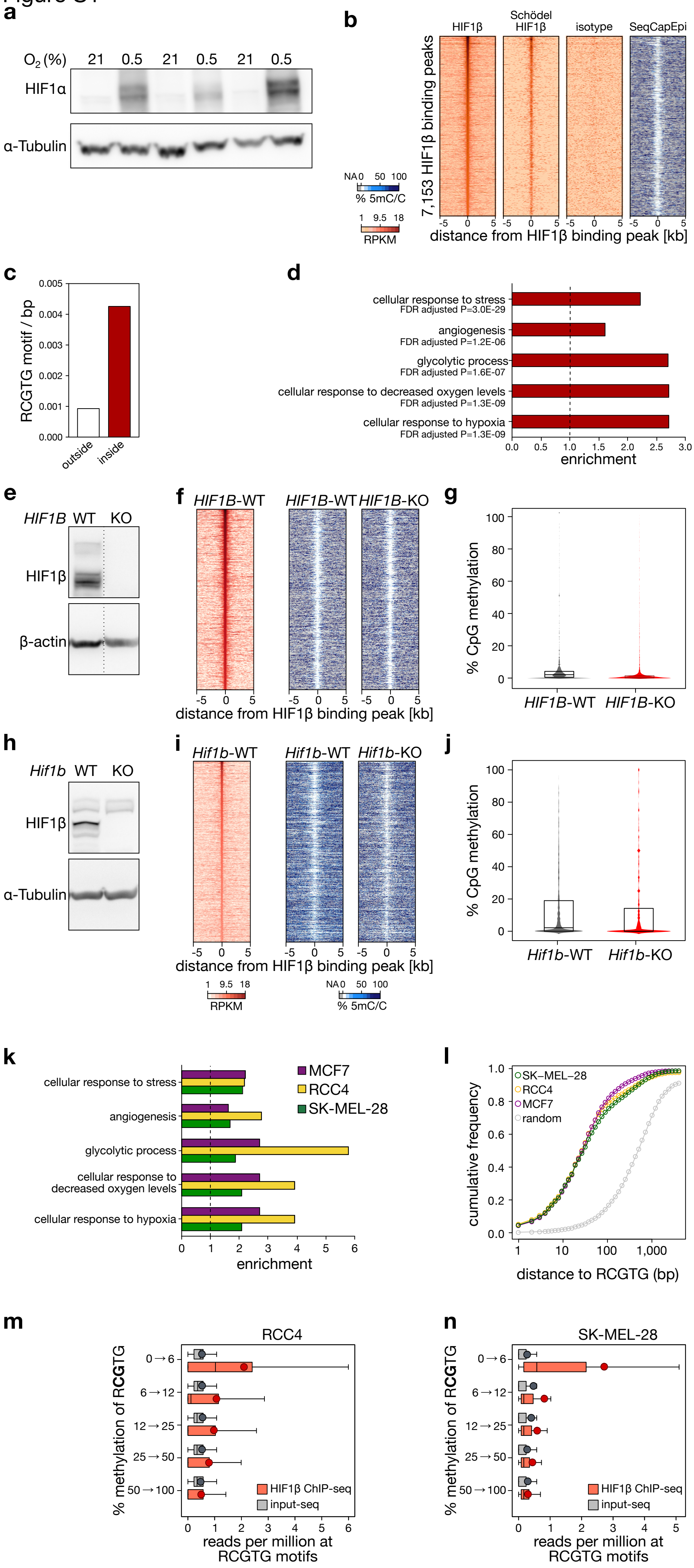

a
O2 (%) 21 0.5 21 0.5 21 0.5
HIF1α
α-Tubulin
b
HIF1β
Schödel HIF1β
isotype
SeqCapEpi
7,153 HIF1β binding peaks
distance from HIF1β binding peak [kb]
% 5mC/C
RPKM
c
RCGTG motif / bp
outside
inside
d
cellular response to stress
FDR adjusted P=3.0E-29
angiogenesis
FDR adjusted P=1.2E-06
glycolytic process
FDR adjusted P=1.6E-07
cellular response to decreased oxygen levels
FDR adjusted P=1.3E-09
cellular response to hypoxia
FDR adjusted P=1.3E-09
enrichment
e
HIF1B WT KO
HIF1β
β-actin
f
HIF1B-WT HIF1B-WT HIF1B-KO
distance from HIF1β binding peak [kb]
g
% CpG methylation
HIF1B-WT HIF1B-KO
h
Hif1b WT KO
HIF1β
α-Tubulin
i
Hif1b-WT Hif1b-WT Hif1b-KO
distance from HIF1β binding peak [kb]
RPKM
% 5mC/C
j
% CpG methylation
Hif1b-WT Hif1b-KO
k
cellular response to stress
angiogenesis
glycolytic process
cellular response to decreased oxygen levels
cellular response to hypoxia
MCF7
RCC4
SK-MEL-28
enrichment
l
SK–MEL–28
RCC4
MCF7
random
cumulative frequency
distance to RCGTG (bp)
m
RCC4
% methylation of RCGTG
0 → 6
6 → 12
12 → 25
25 → 50
50 → 100
HIF1β ChIP-seq
input-seq
reads per million at RCGTG motifs
n
SK-MEL-28
% methylation of RCGTG
HIF1β ChIP-seq
input-seq
reads per million at RCGTG motifs

Fig S1 | HIF1β peaks in MCF7 cells under 0.5% O_2

(**a**) *(top)* Immunoblot analysis of HIF1α in MCF7 cells exposed for 24 hours to 21% or 0.5% O_2. (**b**) Comparison of the 7,153 HIF1β peaks detected in this study to a previously published dataset of ChIP-seq for HIF1β. (**c**) Frequency per basepair of the RCGTG motif inside and outside HIF1β peaks. On average, 4.6 HIF consensus sequences (RCGTG) are found in each HIF1β peak (average length 1,088 bps), whereas in the rest of the genome, 0.98 HIF consensus sequences (RCGTG) are found every 1,088 bps (Fisher exact test $P<2.2^{-16}$). (**d**) Ontology analysis of genes associated with the 7,153 HIF1β peaks detected in MCF7 cells by ChIP-seq for HIF1β. (**e**) Immunoblot analysis of HIF1β in MCF7 cells that are wild-type (WT) or knockout (KO) for HIF1β. (**f-g**) Heatmaps of HIF1β binding for WT MCF7 cells and of DNA methylation for WT and *HIF1B*-KO MCF7 cells, at regions surrounding the HIF1β ChIP-seq peak summit in WT MCF7 cells (±5 kb) (*f*), and violin and boxplots of methylation at the HIF1β ChIP-seq peak summit (±100 bps) for WT and *HIF1B*-KO MCF7 cells (*g*). Depicted are the data from the 4,794 regions having >30-fold methylation coverage for both genotypes (WT and KO) in the 100 bps flanking the binding summit, as assessed using SeqCapEpi BS-seq. HIF1β binding was assessed after 16 hours of 0.5% O_2 (hypoxia), and DNA methylation under 21% O_2 (normoxia). (**h**) Immunoblot analysis of HIF1β in mESCs that are wild-type (WT) or knockout (KO) for *Hif1b*. (**i, j**) Heatmaps of HIF1β binding for ESCs and of DNA methylation for murine WT and *Hif1b*-KO ESCs, at regions surrounding the HIF1β ChIP-seq peak summit in WT ESCs (±5 kb) (*i*), and violin and boxplots of methylation at the HIF1β ChIP-seq peak summit (±100 bps) for WT and *Hif1b*-KO ESCs (*j*). Depicted are the data from the 1,644 regions having >30-fold methylation-coverage for both genotypes (WT and KO) in the 100 bps flanking the binding summit, as assessed using whole-genome BS-seq. HIF1β binding was assessed after 16 hours of 0.5% O_2 (hypoxia), and DNA methylation under 21% O_2 (normoxia). (**k**) Ontology analysis of genes associated with HIF1β peaks in 3 cell lines (SK-MEL-28, green; RCC4, yellow; MCF7, purple). (**l**) Cumulative frequency of distance to the nearest RCGTG motif, for HIF1β binding peak summits detected in 3 cell lines and for a random set of genomic positions. (**m-n**) Sequencing read depth of HIF1β ChIP and its input, at all RCGTG sequences in RCC4 (*m*) and SK-MEL-28 (*n*) cells, stratified for methylation at the CG in the core R<u>CG</u>TG sequence. Shown are data for all RCGTG sequences in the human genome for which >10× coverage was obtained after SeqCapEpi BS-seq.

Figure S2

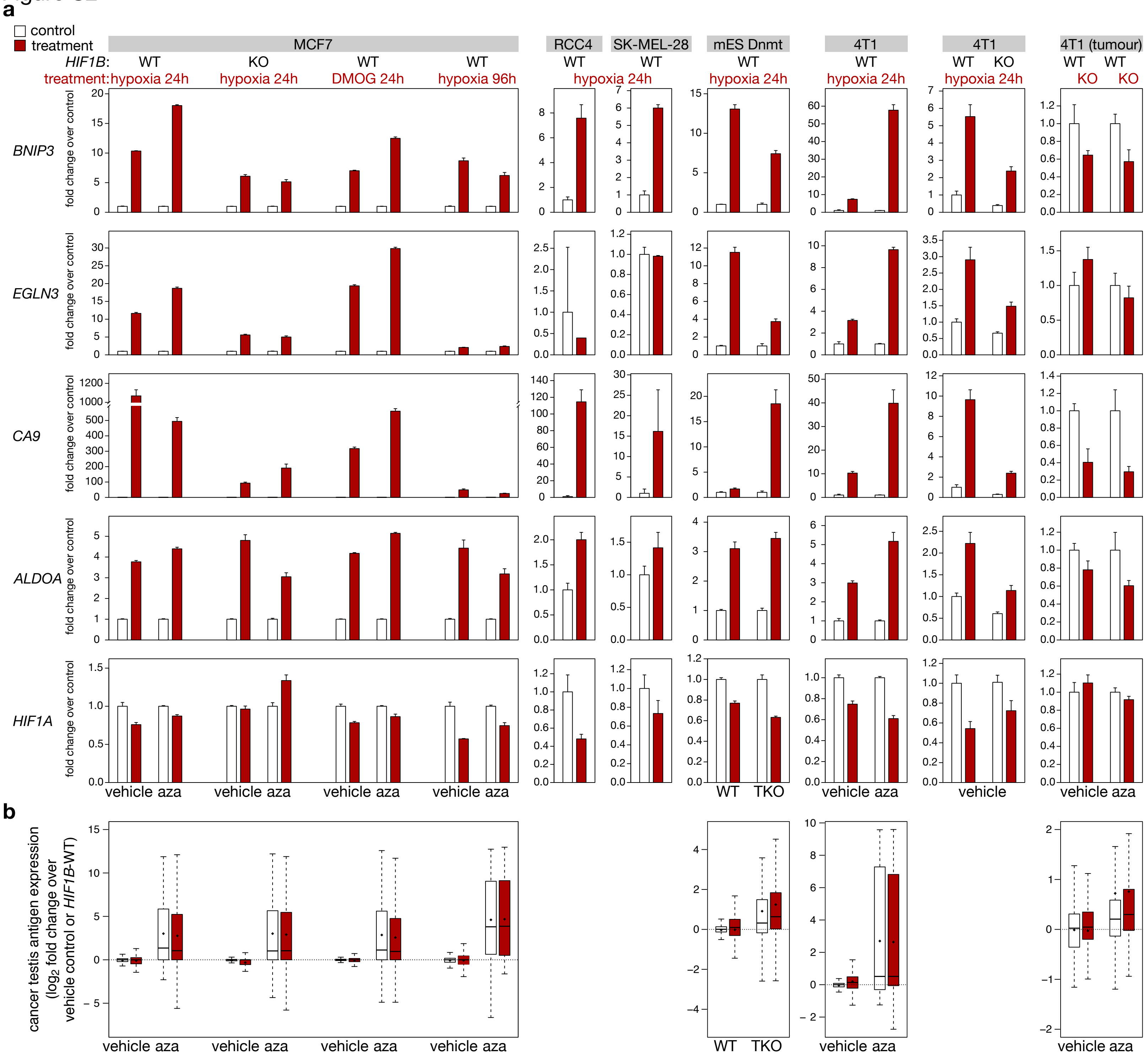

a
control
treatment
MCF7
RCC4
SK-MEL-28
mES Dnmt
4T1
4T1
4T1 (tumour)
HIF1B:
WT
KO
WT
WT
treatment:
hypoxia 24h
hypoxia 24h
DMOG 24h
hypoxia 96h
hypoxia 24h
hypoxia 24h
hypoxia 24h
hypoxia 24h
KO
KO
BNIP3
EGLN3
CA9
ALDOA
HIF1A
fold change over control
vehicle aza
WT TKO
vehicle
b
cancer testis antigen expression (log2 fold change over vehicle control or HIF1B-WT)

Fig. S2 | Expression of hypoxia genes and cancer testis antigens

(**a**) mRNA expression of hypoxia-induced genes in human and mouse cell lines and in 4T1 tumours. Shown is the average fold change of transcript per million over the appropriate control. (**b**) mRNA expression of cancer testis antigens in human cell lines, in 4T1 cells and mESCs exposed to hypoxia, DMOG, aza or deficient for the indicated gene (TKO for *Dnmt*). Shown are boxplots of $\log_2$ transcripts per million normalized over the proper control.

Figure S3

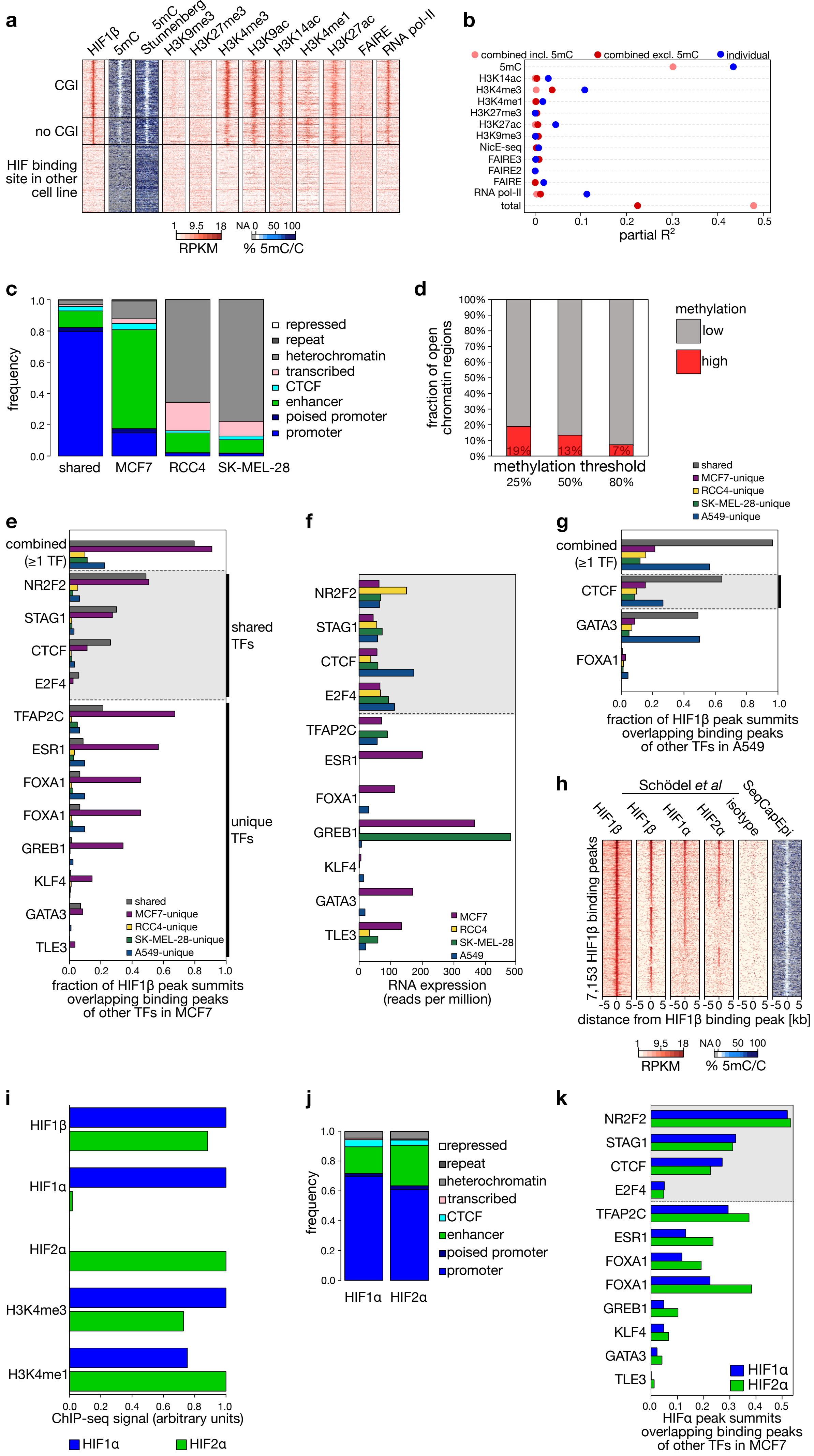

a
HIF1β 5mC 5mC Stunnenberg H3K9me3 H3K27me3 H3K4me3 H3K9ac H3K14ac H3K4me1 H3K27ac FAIRE RNA pol-II
CGI
no CGI
HIF binding site in other cell line
RPKM
% 5mC/C
b
combined incl. 5mC
combined excl. 5mC
individual
partial R^2
c
frequency
shared MCF7 RCC4 SK-MEL-28
repressed
repeat
heterochromatin
transcribed
CTCF
enhancer
poised promoter
promoter
d
fraction of open chromatin regions
methylation threshold
25% 50% 80%
methylation
low
high
e
combined (≥1 TF)
shared TFs
unique TFs
fraction of HIF1β peak summits overlapping binding peaks of other TFs in MCF7
f
RNA expression (reads per million)
g
fraction of HIF1β peak summits overlapping binding peaks of other TFs in A549
h
Schödel et al
SeqCapEpi
isotype
7,153 HIF1β binding peaks
distance from HIF1β binding peak [kb]
i
ChIP-seq signal (arbitrary units)
HIF1α
HIF2α
j
frequency
k
HIFα peak summits overlapping binding peaks of other TFs in MCF7

Fig. S3 | Cell-type-specific HIF1β binding

(**a**) Patterns of DNA methylation and of the indicated chromatin modifications in MCF7 cells, at HIF1β binding sites detected in MCF7 cells at CpG islands (*top*), not at CpG islands (*middle*) or in SK-MEL-28 and/or RCC4 cells but not MCF7 (*bottom*). DNA methylation was determined using SeqCapEpi BS-seq or WGBS; occupancy of chromatin modifications was determined using the ENCODE project. (**b**) Estimates of the contribution of individual epigenetic marks in predicting the presence of HIF1β binding peaks, as determined by partial R^2 analysis of linear regression models. Shown are the results of a linear model using all available marks, a linear model using all available marks but not DNA methylation, and the results of linear models using each modification in an individual analysis. All 3 available FAIRE-seq datasets[68, 69] show similar results. (**c**) Functional genome annotation of MCF7 cells using ChromHMM, at shared and cell-type-specific HIF1β binding peaks. (**d**) Fraction of regions in MCF7 cells showing high *versus* low methylation in regions of open chromatin. 10,805 regions of open chromatin that showed high GpC methylation levels were assessed (>10-fold coverage) for their CpG methylation. Indicated are the fraction of these 10,805 regions showing at least 25%, 50% or 80% CpG methylation. GCG trinucleotides were not considered in these analyses. (**e**) Fraction of HIF1β binding peaks, shared between or unique for a cell line, that overlaps with other TF binding sites detected in MCF7 cells. Note how HIF1β binding peaks unique to MCF7 cells (*purple*) predominantly overlap with TFs uniquely expressed in MCF7 cells (TFASP2C, ESR1, …), while HIF1β binding peaks shared between cell lines (*dark grey*) more frequently overlap with commonly expressed TFs (NR2F2, STAG1, …). (**f**) mRNA expression level of indicated transcription factors in each cell line, as determined using RNA-seq. These data enable stratification of TFs into those that are shared in expression (*light grey box*) and those that are uniquely expressed (*white box*). (**g**) Fraction of HIF1β binding peaks, shared between or unique for a cell line, that overlaps with other transcription factor binding sites detected in A549 cells. Note how HIF1β binding peaks unique to A549 cells (*blue*) predominantly overlap with TFs expressed in A549 cells (GATA3 and FOXA1), while HIF1β binding peaks shared between cell lines (*dark grey*) more frequently overlap with binding of CTCF, a commonly expressed TF. (**h**) Comparison of the HIF1β peaks detected to a previously published dataset of ChIP-seq for HIF1β, HIF1α and HIF2α. (**i**) ChIP-seq signal for the indicated epitopes at the 200 bps surrounding the HIF1β peak summits that are uniquely co-bound by HIF1α or HIF2α. (**j**) Functional genome annotation of MCF7 cells using ChromHMM, at HIF1β binding peaks that are uniquely co-bound by HIF1α or HIF2α. (**k**) Fraction of HIF1β binding peaks, uniquely co-bound by HIF1α or HIF2α, that overlaps with other transcription factor binding sites detected in MCF7 cells.

Figure S4

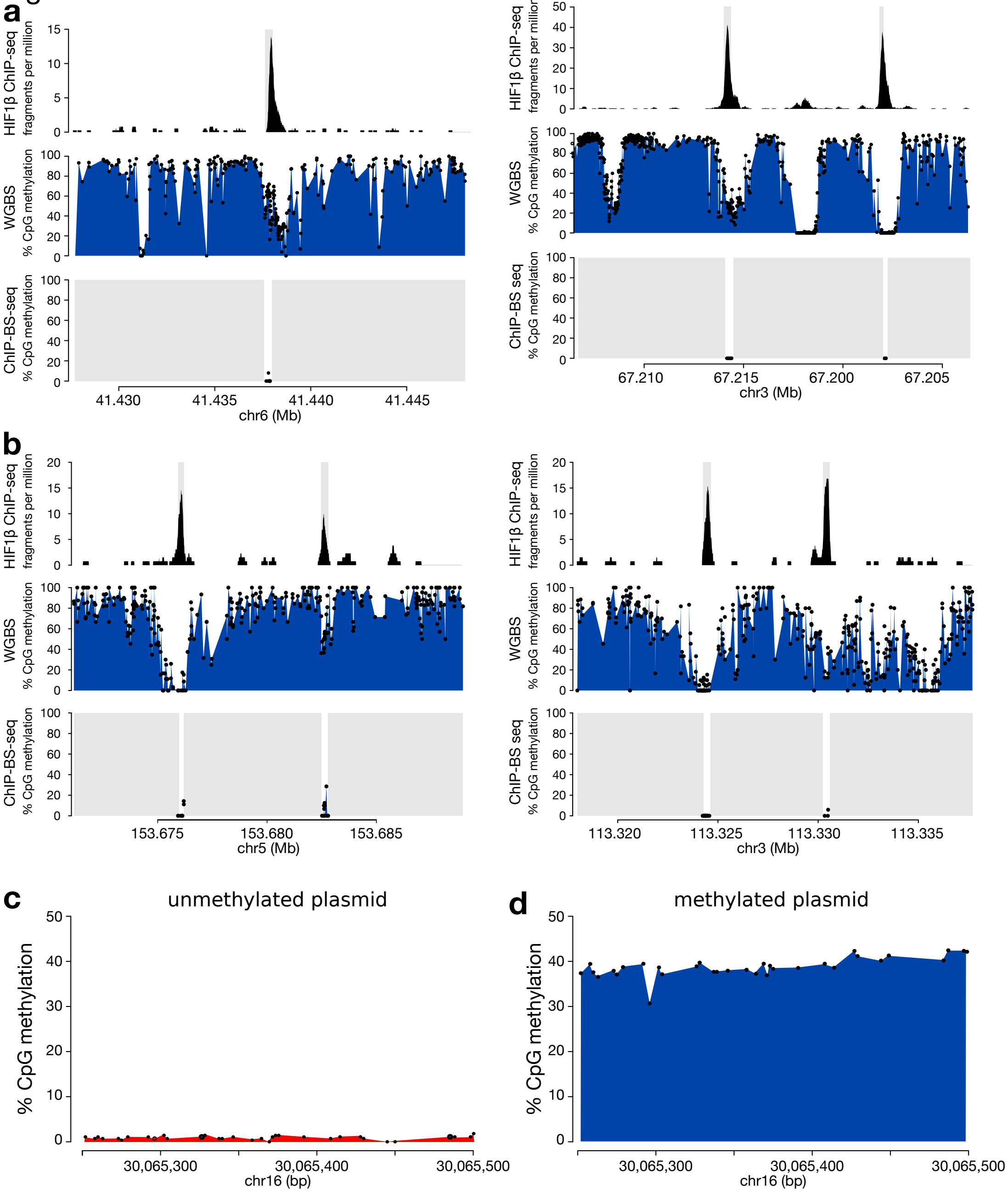

a
HIF1β ChIP-seq fragments per million
WGBS % CpG methylation
ChIP-BS-seq % CpG methylation
41.430
41.435
41.440
41.445
chr6 (Mb)
HIF1β ChIP-seq fragments per million
WGBS % CpG methylation
ChIP-BS seq % CpG methylation
67.210
67.215
67.200
67.205
chr3 (Mb)
b
HIF1β ChIP-seq fragments per million
WGBS % CpG methylation
ChIP-BS-seq % CpG methylation
153.675
153.680
153.685
chr5 (Mb)
HIF1β ChIP-seq fragments per million
WGBS % CpG methylation
ChIP-BS seq % CpG methylation
113.320
113.325
113.330
113.335
chr3 (Mb)
c
unmethylated plasmid
% CpG methylation
30,065,300
30,065,400
30,065,500
chr16 (bp)
d
methylated plasmid
% CpG methylation
30,065,300
30,065,400
30,065,500
chr16 (bp)

Fig. S4 | DNA methylation directly repels HIF1β binding

(**a**) Example plots of regions on chromosomes 6 and 16, showing HIF1β ChIP-seq read depths, and methylation levels as determined by whole-genome BS-seq and HIF1β ChIP-BS-seq in MCF7 cells. Coordinates: human genome build hg19. Grey boxes in HIF1β ChIP-BS-seq tracks indicate regions where insufficient sequences were recovered. (**b**) As in (*a*), but in murine *Tet*-TKO ESCs, and for regions on chromosomes 5 and 3. Coordinates follow genome build Mm10. In each example plot, HIF1β peaks (fragment per million) are shown on top; percentage of DNA methylation (calculated in bulk normoxic cells by WGBS) at the same position of the HIF1β peaks is shown in the middle; percentage of DNA methylation of the immunoprecipitated DNA fragment from HIF1β ChIP-BS-seq is shown at the bottom. (**c-d**) Methylation levels of a human DNA fragment inserted in mESCs, as detected using amplicon bisulfite sequencing. A human HIF binding site (chr16: 30,065,212-30,065,711 on hg38) was cloned between 2 L1 *Lox* sites and *in vitro* methylated (*d*) or not (*c*) prior to insertion into mESCs transformed to contain an L1 *Lox*-flanked thymidine kinase.

Figure S5

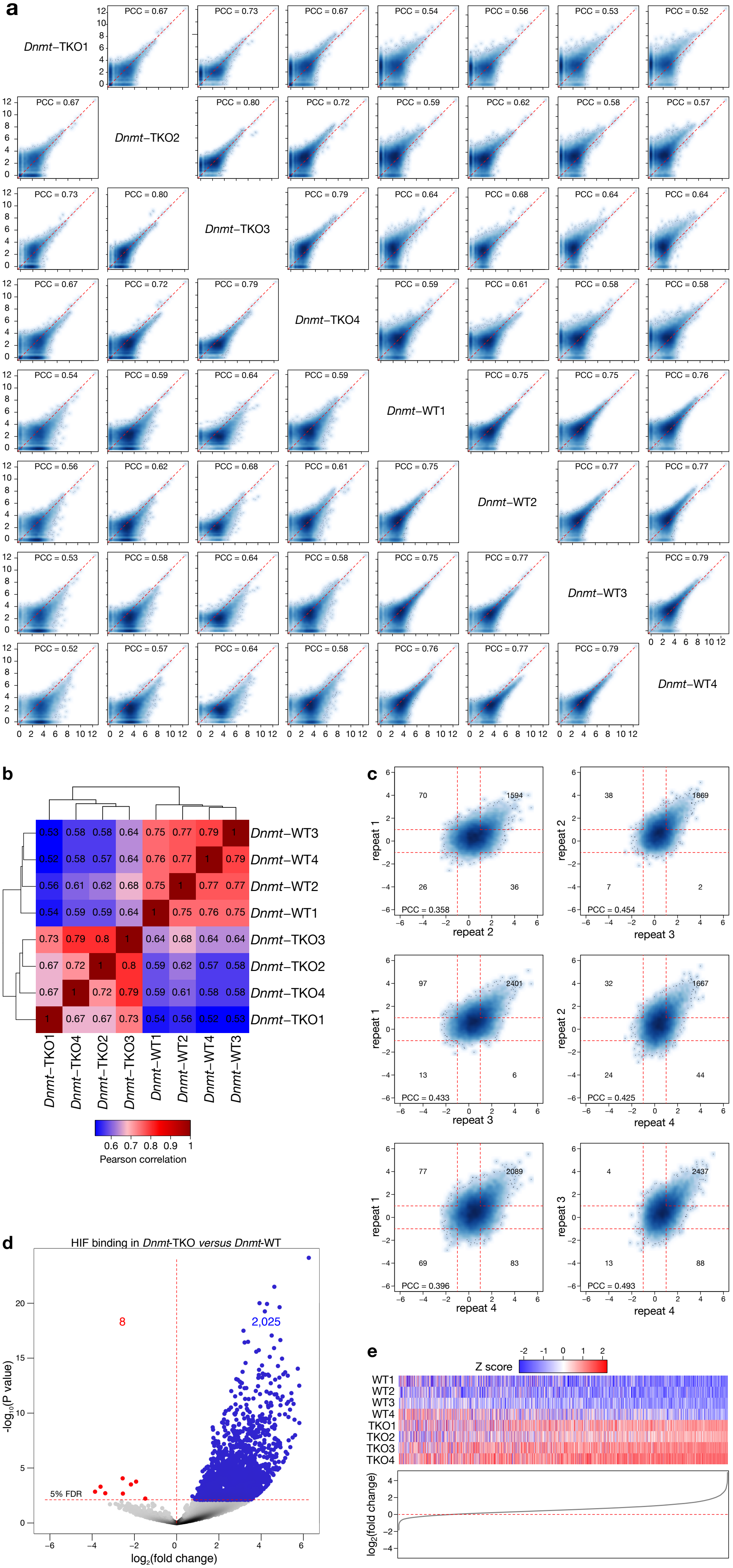

a
Dnmt–TKO1
Dnmt–TKO2
Dnmt–TKO3
Dnmt–TKO4
Dnmt–WT1
Dnmt–WT2
Dnmt–WT3
Dnmt–WT4
PCC = 0.67
PCC = 0.73
PCC = 0.54
PCC = 0.56
PCC = 0.53
PCC = 0.52
PCC = 0.80
PCC = 0.72
PCC = 0.59
PCC = 0.62
PCC = 0.58
PCC = 0.57
PCC = 0.79
PCC = 0.64
PCC = 0.68
PCC = 0.61
PCC = 0.75
PCC = 0.76
PCC = 0.77
b
Pearson correlation
0.6 0.7 0.8 0.9 1
c
repeat 1
repeat 2
repeat 3
repeat 4
PCC = 0.358
PCC = 0.454
PCC = 0.433
PCC = 0.425
PCC = 0.396
PCC = 0.493
d
HIF binding in Dnmt-TKO versus Dnmt-WT
8
2,025
5% FDR
-log10(P value)
log2(fold change)
e
Z score
WT1
WT2
WT3
WT4
TKO1
TKO2
TKO3
TKO4
log2(fold change)

Fig. S5 | Quality control of the ChIP-seq replicates

(**a**) Smooth-scatter heatmaps for each experimental HIF1β ChIP-seq repeat, showing pairwise comparisons of the read depth ($\log_2$(RPKM+1)) at a peak set representing the union of peaks detected in WT and *Dnmt*-TKO cells. (**b**) Hierarchically clustered heatmap of Pearson correlations between the $\log_2$ read counts of all replicates for a peak set representing the union of all peaks in WT and *Dnmt*-TKO cells. Note how Pearson correlations between replicates of the same genetic background are higher than the correlations across the two genetic background. (**c**) A merged peak set (union of all peaks in WT and *Dnmt*-TKO cells) was generated, and the difference between the ChIP-seq signals ($\log_2$(RPKM+1) of these peaks of *Dnmt*-TKO and WT), was calculated for each of the 4 repeats. These repeats are here compared with a smooth-scatter heatmap. Also indicated are the associated Pearson Correlation Coefficients (PCC), as well as the number of peaks showing >2-fold change between both repeats. (**d**) Results of EdgeR analysis of differences in ChIP-seq signal strength between the 4 HIF1β ChIP-seq replicates. Shown are the significant differences between the 4 wild-type and 4 *Dnmt*-TKO (TKO, red) replicates. Dots colored in red and blue indicate peaks showing respectively decreased and increased binding in *Dnmt*-TKO cells *versus* WT cells. (**e**) Heatmaps showing the relative signal of the 4 HIF1β ChIP-seq data replicates, ranked by their mean fold change (quantified below). Shown are 4 wild-type (WT, blue) and 4 *Dnmt*-TKO (TKO, red) replicates.

Figure S6

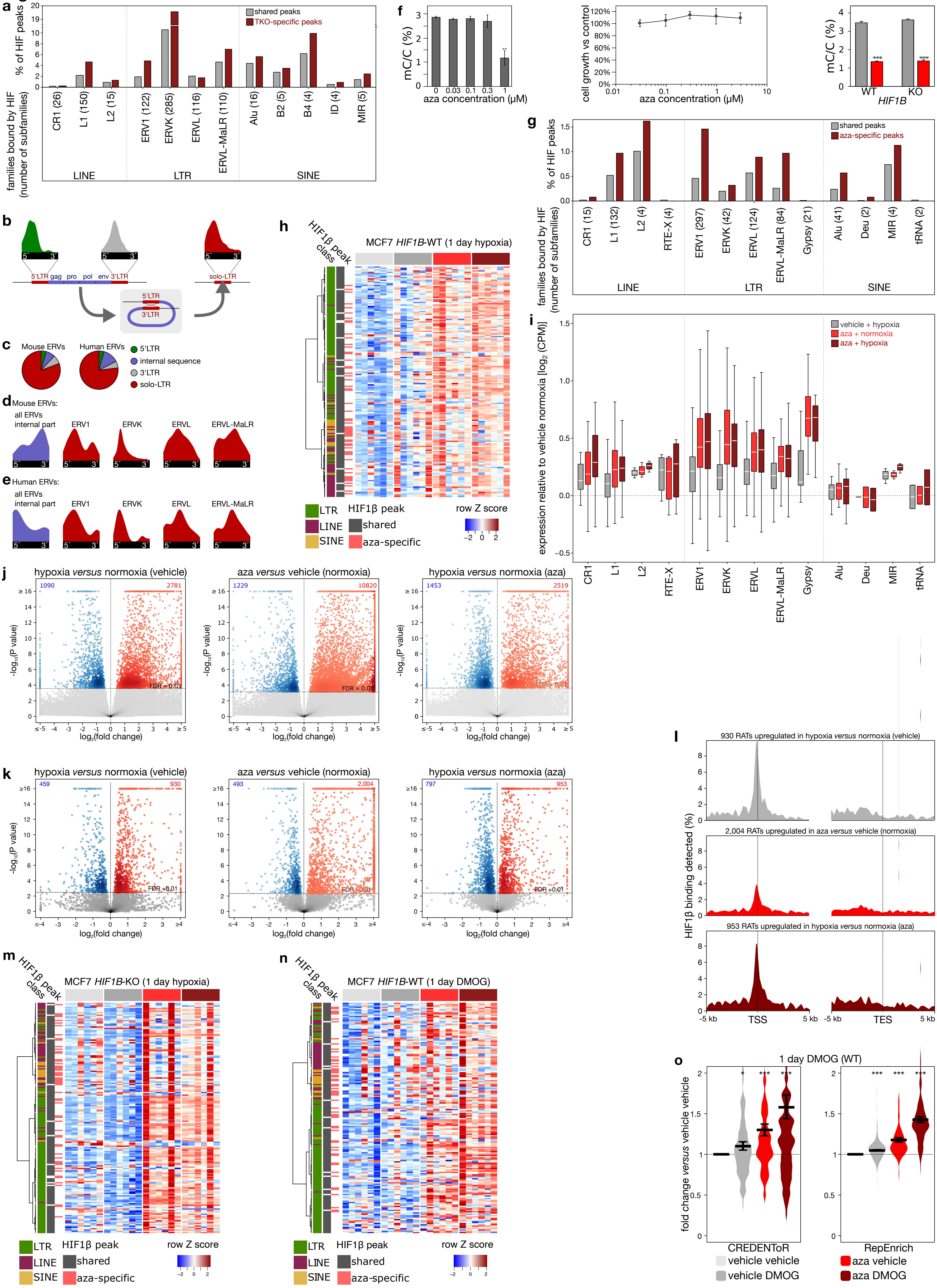

a
% of HIF peaks
shared peaks
TKO-specific peaks
families bound by HIF (number of subfamilies)
CR1 (26)
L1 (150)
L2 (15)
ERV1 (122)
ERVK (285)
ERVL (116)
ERVL-MaLR (110)
Alu (16)
B2 (5)
B4 (4)
ID (4)
MIR (5)
LINE
LTR
SINE
b
5'LTR gag pro pol env 3'LTR
solo-LTR
c
Mouse ERVs
Human ERVs
5'LTR
internal sequence
3'LTR
solo-LTR
d
Mouse ERVs:
all ERVs internal part
ERV1
ERVK
ERVL
ERVL-MaLR
e
Human ERVs:
all ERVs internal part
ERV1
ERVK
ERVL
ERVL-MaLR
f
mC/C (%)
aza concentration (μM)
cell growth vs control
WT
KO
HIF1B
g
% of HIF peaks
shared peaks
aza-specific peaks
families bound by HIF (number of subfamilies)
CR1 (15)
L1 (132)
L2 (4)
RTE-X (4)
ERV1 (297)
ERVK (42)
ERVL (124)
ERVL-MaLR (84)
Gypsy (21)
Alu (41)
Deu (2)
MIR (4)
tRNA (2)
LINE
LTR
SINE
h
HIF1β peak class
MCF7 HIF1B-WT (1 day hypoxia)
LTR
LINE
SINE
HIF1β peak
shared
aza-specific
row Z score
i
expression relative to vehicle normoxia [log₂ (CPM)]
vehicle + hypoxia
aza + normoxia
aza + hypoxia
CR1
L1
L2
RTE-X
ERV1
ERVK
ERVL
ERVL-MaLR
Gypsy
Alu
Deu
MIR
tRNA
j
hypoxia versus normoxia (vehicle)
aza versus vehicle (normoxia)
hypoxia versus normoxia (aza)
1090
2781
1229
10820
1453
2519
-log₁₀(P value)
log₂(fold change)
FDR = 0.01
k
hypoxia versus normoxia (vehicle)
aza versus vehicle (normoxia)
hypoxia versus normoxia (aza)
459
930
493
2,004
797
953
-log₁₀(P value)
log₂(fold change)
FDR = 0.01
l
930 RATs upregulated in hypoxia versus normoxia (vehicle)
2,004 RATs upregulated in aza versus vehicle (normoxia)
953 RATs upregulated in hypoxia versus normoxia (aza)
HIF1β binding detected (%)
-5 kb
TSS
5 kb
TES
m
HIF1β peak class
MCF7 HIF1B-KO (1 day hypoxia)
LTR
LINE
SINE
HIF1β peak
shared
aza-specific
row Z score
n
HIF1β peak class
MCF7 HIF1B-WT (1 day DMOG)
LTR
LINE
SINE
HIF1β peak
shared
aza-specific
row Z score
o
1 day DMOG (WT)
fold change versus vehicle vehicle
CREDENToR
RepEnrich
vehicle vehicle
vehicle DMOG
aza vehicle
aza DMOG

Fig. S6 I HIF binds retrotransposons in demethylated genomes

(**a**) % of shared or *Dnmt*-TKO-specific (TKO-specific) HIF1β binding peaks in ESCs that overlap retrotransposons, grouped by retrotransposon class and family. Families bound less than 3 times by HIF1β (Dong-R4, Jockey, Penelope, RTE-X, Gypsy, 5S-Deu-L2, tRNA, tRNA-Deu) or containing unclassifiable subfamilies are not shown. The number of retrotransposon subfamilies involved is indicated in brackets. (**b**) HIF binding at long terminal repeats (LTRs, red) and internal sequences (blue). A solo-LTRs arise through non-allelic homologous recombination of the 5' and 3' LTRs (*bottom*). Show are (*top*) the HIF binding sites in murine ERVK LTRs that are at the 5' or 3' end of the ERVK-internal sequence, or that are solitary ("solo-LTR"). As can be appreciated, these have a similar distribution of HIF binding sites. (**c**) Fraction of HIF binding sites in 5', 3' or solo LTRs or in internal sequences, and this in in murine ES cells (*left*) and MCF7 cells (*right*). (**d**-**e**) Distribution of HIF binding at ERV internal sequences (all combined, *blue*) and at ERV1, ERVK, ERVL and ERVL-MaLR LTRs (*red*) in murine ES cells (*d*) and MCF7 cells (*e*). LTRs are grouped by subfamily, irrespective of whether they are 5', 3' or solo LTRs. As the total number of HIF binding sites in the internal sequence was too low for reliably generating density plots, we merged all subfamilies. (**f**) Validations of aza treatment. 5mC content of DNA *(left)* and survival (*middle*) of WT MCF7 cells, exposed to various concentrations of 5-aza-dC, and (*right*) 5mC content of DNA from WT and *HIF1B*-KO MCF7 cells, exposed to 5-aza-dC (aza; 1 μM, red) or DMSO (control, grey) for 3 cell cycles, as determined using LC/MS. (**g**) As in (*a*), but for HIF1β binding peaks that are shared between vehicle- and aza-treated MCF7 cells, or specific to aza-treated MCF7 cells. (**h**) Heatmap showing expression of 176 retrotransposon subfamilies bound by HIF1β in hypoxic and normoxic MCF7 cells exposed to vehicle (DMSO) or aza. Expression was determined using RepEnrich (n=6 per treatment condition). (**i**) Differential expression of 13 retrotransposon families bound by HIF1β. Boxplots show average read counts from RNA-seq analyses of vehicle- and aza-treated MCF7 cells exposed for 24 hours to normoxia or hypoxia (resp. 21% and 0.5% O_2), as determined using RepEnrich (n=6 per condition). Families not bound by HIF1β or containing unclassifiable subfamilies are not shown. (**j**) Volcano plots showing differential expression of retrotransposon loci, as determined by SQuIRE analysis of MCF7 cells exposed for 4 days to vehicle or aza (1 μM), and for 1 day to hypoxia (0.5% oxygen) or normoxia. Significantly upregulated and downregulated loci (1% FDR, horizontal line) are highlighted in red and blue, respectively, with the number of loci being indicated. (**k**) as in (*j*) but for all cryptic transcripts, also those not bound by HIF1β, using an analysis performed with CREDENToR. (**l**) HIF binding sites detected at the TSS and transcription end site (TES) of cryptic transcripts overexpressed (1% FDR) in MCF7 cells exposed to hypoxia and/or aza. Shown are percentages of 250 bp bins wherein a HIF binding peak was detected. (**m-n**) Heatmap showing the expression of 176 retrotransposon subfamilies bound by HIF1β in *HIF1B*-KO MCF7 cells exposed to hypoxia (0.5% O_2) for 24 hours and treated with vehicle or aza (*m*) and in WT (*HIF1B*-WT) MCF7 cells exposed to dimethyloxalylglycine (DMOG; 2 mM) for 24 hours and treated with vehicle or aza (*n*). Expression was determined using RepEnrich (n=6 per treatment condition). (**o**) Expression of HIF-bound cryptic transcripts (CREDENToR, *left*) and retrotransposon subfamilies (RepEnrich, *right*) relative to vehicle-treated control (vehicle vehicle) in MCF7 cells wild-type (WT) for *HIF1B*. *P<0.05, ***P<0.001 by t-test.

Figure S7

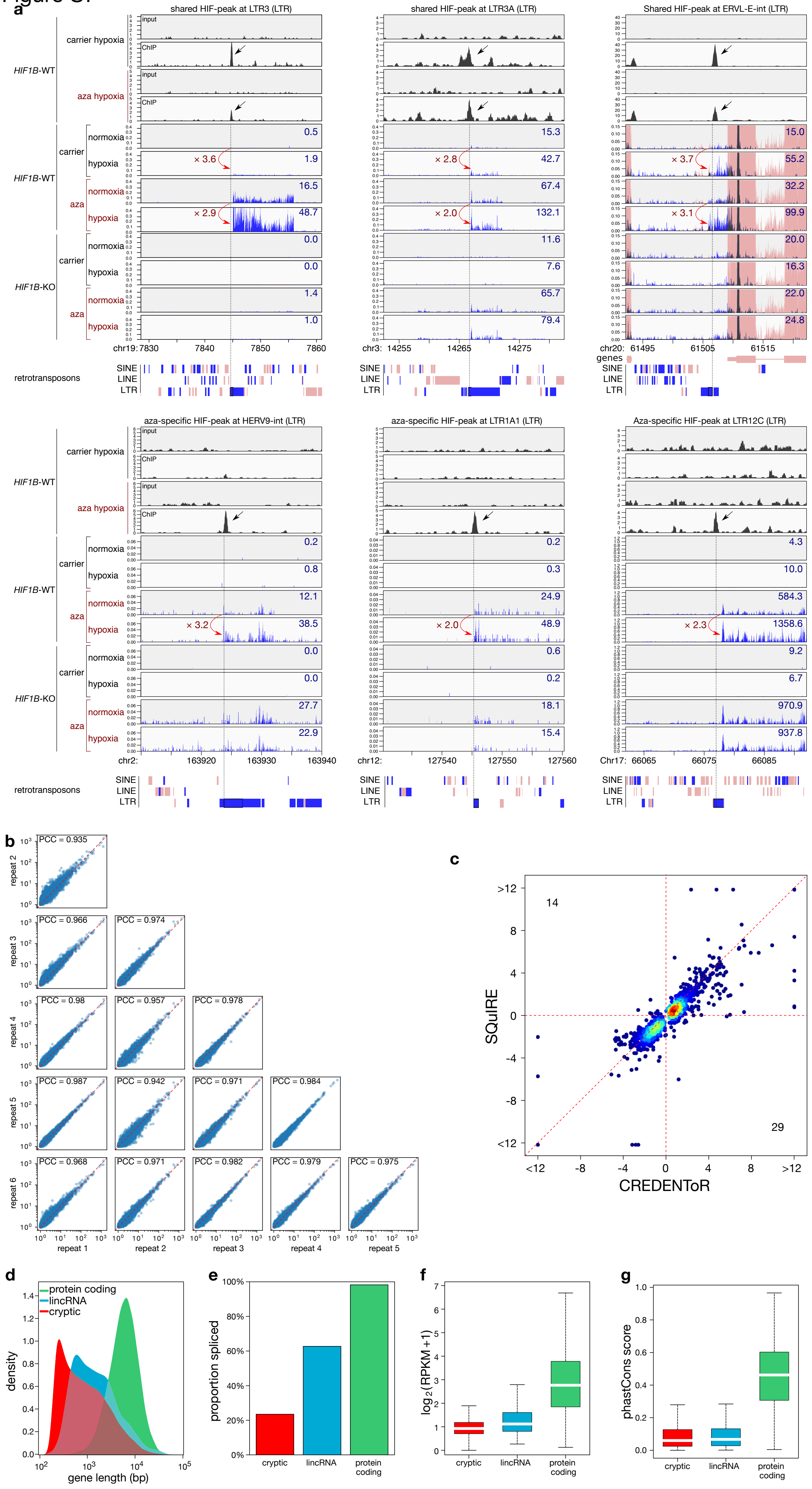
a
shared HIF-peak at LTR3 (LTR)
shared HIF-peak at LTR3A (LTR)
Shared HIF-peak at ERVL-E-int (LTR)
HIF1B-WT
carrier hypoxia
aza hypoxia
input
ChIP
carrier
aza
normoxia
hypoxia
HIF1B-KO
0.5
1.9
16.5
48.7
0.0
0.0
1.4
1.0
× 3.6
× 2.9
15.3
42.7
67.4
132.1
11.6
7.6
65.7
79.4
× 2.8
× 2.0
15.0
55.2
32.2
99.9
20.0
16.3
22.0
24.8
× 3.7
× 3.1
chr19: 7830
7840
7850
7860
chr3: 14255
14265
14275
chr20: 61495
61505
61515
genes
retrotransposons
SINE
LINE
LTR
aza-specific HIF-peak at HERV9-int (LTR)
aza-specific HIF-peak at LTR1A1 (LTR)
Aza-specific HIF-peak at LTR12C (LTR)
0.2
0.8
12.1
38.5
0.0
0.0
27.7
22.9
× 3.2
0.2
0.3
24.9
48.9
0.6
0.2
18.1
15.4
× 2.0
4.3
10.0
584.3
1358.6
9.2
6.7
970.9
937.8
× 2.3
chr2:
163920
163930
163940
chr12:
127540
127550
127560
Chr17: 66065
66075
66085
b
PCC = 0.935
PCC = 0.966
PCC = 0.974
PCC = 0.98
PCC = 0.957
PCC = 0.978
PCC = 0.987
PCC = 0.942
PCC = 0.971
PCC = 0.984
PCC = 0.968
PCC = 0.971
PCC = 0.982
PCC = 0.979
PCC = 0.975
repeat 1
repeat 2
repeat 3
repeat 4
repeat 5
repeat 6
c
SQuIRE
CREDENToR
14
29
>12
<12
d
protein coding
lincRNA
cryptic
density
gene length (bp)
e
proportion spliced
cryptic
lincRNA
protein coding
f
log2(RPKM+1)
g
phastCons score

Fig. S7 I Examples of cryptic transcripts upregulated by HIF1β

(**a**) Examples of HIF1β binding near cryptic transcripts (*top 4 tracks*) and the associated induction of RNA expression (*bottom 8 tracks*). Shown are 3 regions of 30 kb surrounding a HIF1β binding peak that is shared between vehicle-treated and aza-treated MCF7 cells (*top*) and 3 30 kb regions surrounding a HIF1β binding peak that is specifically detected in aza-treated MCF7 cells (*bottom*). Peaks of interest are indicated with an arrow, quantified expression levels of the cryptic transcripts (RPM; upper right corner), and the associated fold changes in expression (red arrow) are shown. As RNA-seq was directional, reads mapping to the top strand (*red*) or the bottom strand (*blue*) are shown. These reads are derived from the cDNA, and thus represent RNA transcripts derived from the bottom (*red*) or top (*blue*) strand respectively. Overlapping reads from both strands are coloured in dark grey. ChIP-seq was not directional. MCF7 cell genotypes (*HIF1B*-WT or -KO) and treatments (aza or vehicle; hypoxia or normoxia) are indicated on the left. At least 3 of the observed cryptic transcripts consist of multiple repeat elements and are thus characterized by read-through of the individual repeat. X-axis: chromosome positions are annotated in kb according to human genome build hg19; Y-axis: reads per kb per million reads. (**b**) Reproducibility of CREDENToR in determining transcript levels (*left*) and hypoxia-induced changes (*right*) in 6 replicates of MCF7 cells exposed to 1 day of hypoxia. PCC: Pearson Correlation Coefficient. (**c**) Comparison between repeat transcript expression estimates obtained by CREDENToR and SQuIRE. (**d**-**g**) Characterization of key features of the cryptic transcripts detected using CREDENToR, in comparison to annotated long intergenic non-coding RNAs (lincRNAs) and protein coding RNAs. Shown are the transcript lengths (*d*), the fraction of transcripts that are spliced (*e*), the RNA expression (*f*) and the conservation estimated using the average phastCon score per transcript (*d*). RPKM: reads per million reads and per kb.

Figure S8

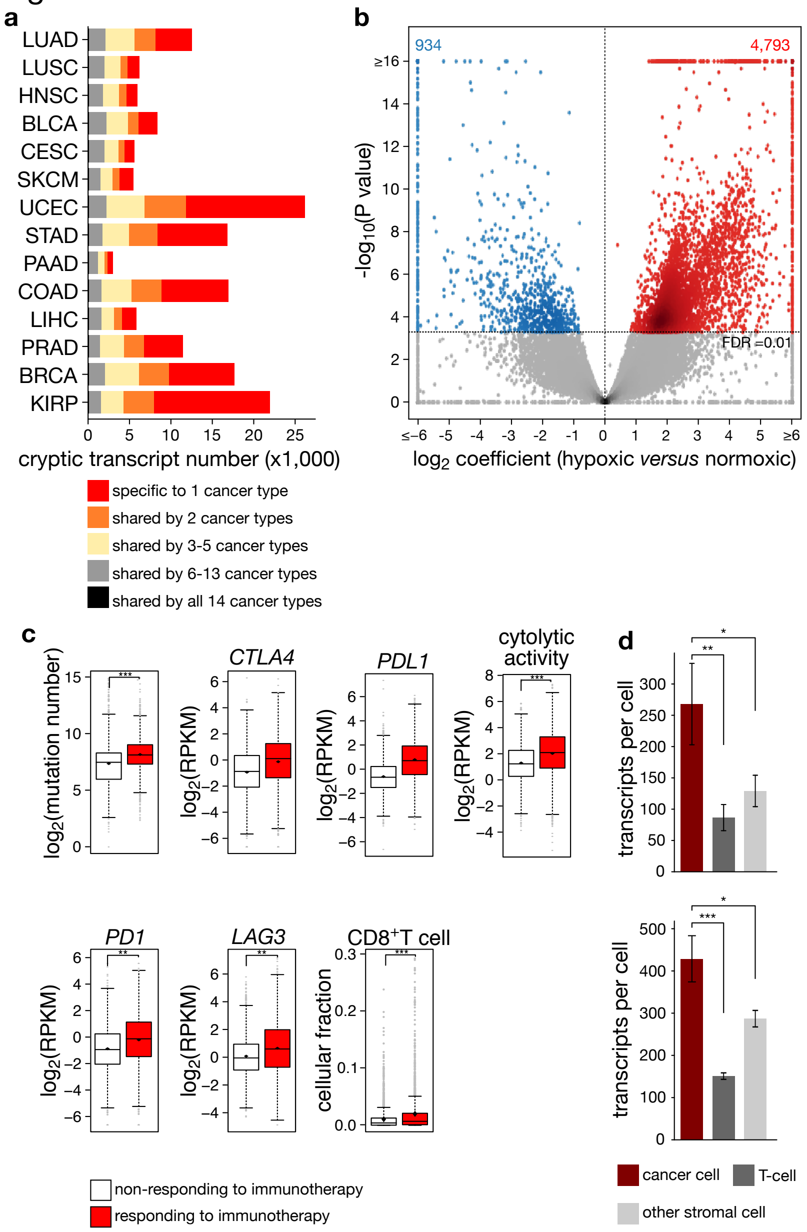
a
LUAD
LUSC
HNSC
BLCA
CESC
SKCM
UCEC
STAD
PAAD
COAD
LIHC
PRAD
BRCA
KIRP
0 5 10 15 20 25
cryptic transcript number (x1,000)
specific to 1 cancer type
shared by 2 cancer types
shared by 3-5 cancer types
shared by 6-13 cancer types
shared by all 14 cancer types
b
934
4,793
-log10(P value)
FDR =0.01
log2 coefficient (hypoxic versus normoxic)
c
log2(mutation number)
CTLA4
PDL1
cytolytic activity
log2(RPKM)
PD1
LAG3
CD8+T cell
cellular fraction
non-responding to immunotherapy
responding to immunotherapy
d
transcripts per cell
cancer cell
T-cell
other stromal cell

Fig. S8 | Cryptic transcript expression in TCGA tumours

(**a**) Recurrent detection of cryptic transcripts in different cancer types profiled in TCGA. Shown are the number of cryptic transcripts detected in 1 cancer type or in different cancer types, as per the legend. (**b**) Volcano plot showing the effect of the interaction between cryptic transcript DNA methylation and tumour oxygenation on the expression of individual cryptic transcripts, tested by DESeq. Positive coefficients represent the cooperative enhancement of cryptic transcript expression in low-methylation, hypoxic conditions. Highlighted in red and blue are significantly positive and negative coefficients (5% FDR) respectively. (**c**) Immunogenicity estimates for TCGA tumours responsive or non-responsive to immunotherapy as described by Turajlik and colleagues[38] (*red and white*). Shown are number of somatic mutations extracted from the TCGA database, mRNA expression of *PDL1*, *PD1* and *LAG3* as $\log_2$ RPKM, cytolytic activity (CYT) defined as the $\log_2$-average (geometric mean) of *GZMA* and *PRF1* expression in RPKM, cell fraction for $CD8^+$ T-cells estimated with respect to the total cells in the sample as defined by quanTIseq (The Cancer Immunome Atlas, TCIA[67]), and cryptic transcript expression as $\log_{10}$ counts per million. **P<0.01 and ***P<0.001 by t-test. (**d**) The number of cryptic transcripts expressed in cancer cells, T-cells and other stromal cells isolated from non-small cell lung tumours (left, n=5) or from breast tumours (right, n=22) and profiled using single-cell RNA-sequencing.[64] Note that this expression dataset was generated using unique molecular identifiers at the 3'-end or 5'-end of poly-adenylated transcripts (resp. left and right panel), enabling the counting of transcripts per cell.

Figure S9

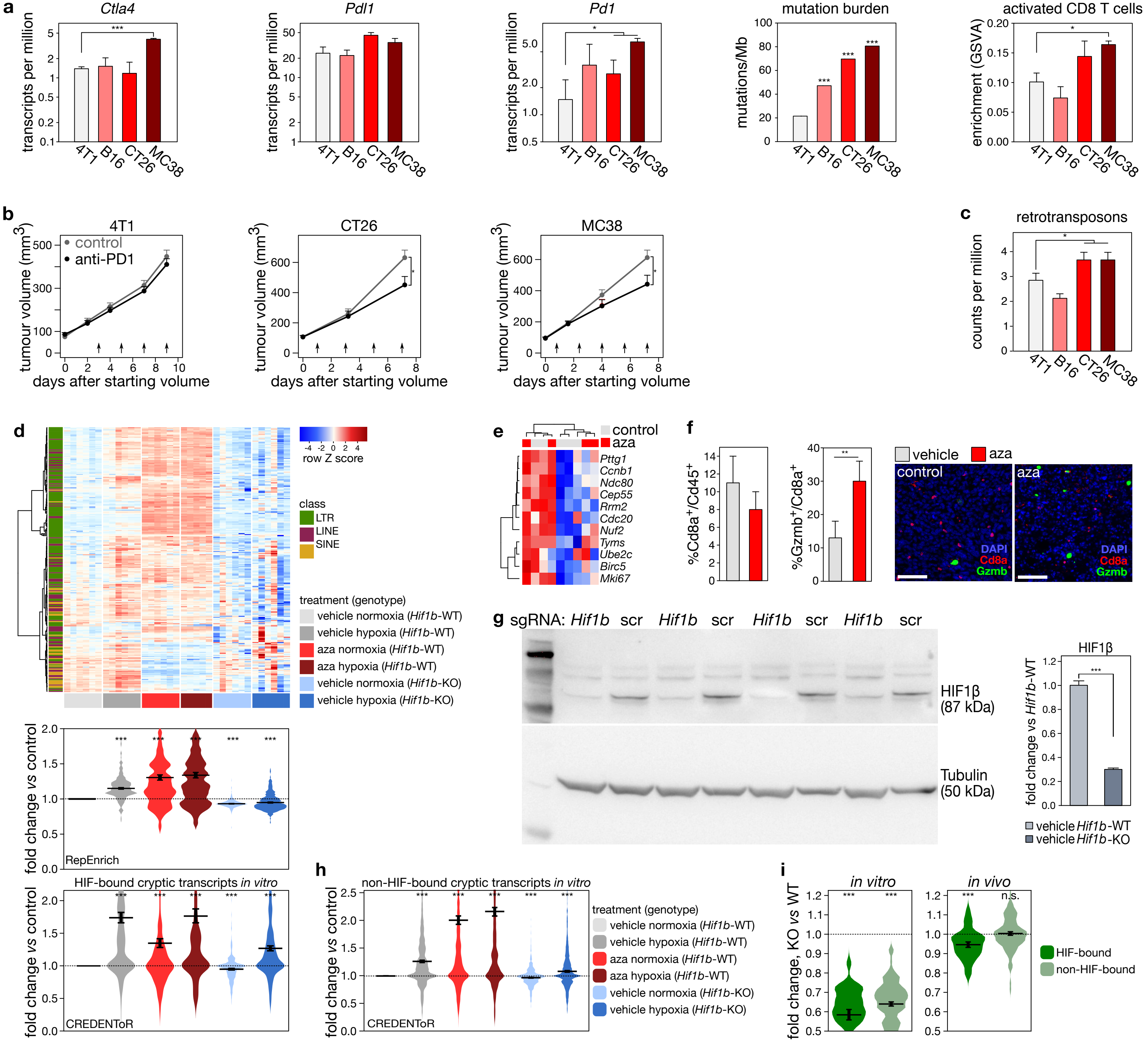

a
Ctla4
Pdl1
Pd1
mutation burden
activated CD8 T cells
transcripts per million
mutations/Mb
enrichment (GSVA)
4T1
B16
CT26
MC38
b
4T1
CT26
MC38
control
anti-PD1
tumour volume (mm3)
days after starting volume
c
retrotransposons
counts per million
d
row Z score
class
LTR
LINE
SINE
treatment (genotype)
vehicle normoxia (Hif1b-WT)
vehicle hypoxia (Hif1b-WT)
aza normoxia (Hif1b-WT)
aza hypoxia (Hif1b-WT)
vehicle normoxia (Hif1b-KO)
vehicle hypoxia (Hif1b-KO)
fold change vs control
RepEnrich
HIF-bound cryptic transcripts in vitro
CREDENToR
e
control
aza
Pttg1
Ccnb1
Ndc80
Cep55
Rrm2
Cdc20
Nuf2
Tyms
Ube2c
Birc5
Mki67
f
%Cd8a+/Cd45+
%Gzmb+/Cd8a+
vehicle
aza
control
aza
DAPI
Cd8a
Gzmb
g
sgRNA: Hif1b scr Hif1b scr Hif1b scr Hif1b scr
HIF1β
(87 kDa)
Tubulin
(50 kDa)
HIF1β
fold change vs Hif1b-WT
vehicle Hif1b-WT
vehicle Hif1b-KO
h
non-HIF-bound cryptic transcripts in vitro
fold change vs control
CREDENToR
treatment (genotype)
vehicle normoxia (Hif1b-WT)
vehicle hypoxia (Hif1b-WT)
aza normoxia (Hif1b-WT)
aza hypoxia (Hif1b-WT)
vehicle normoxia (Hif1b-KO)
vehicle hypoxia (Hif1b-KO)
i
in vitro
in vivo
fold change, KO vs WT
n.s.
HIF-bound
non-HIF-bound

Fig. S9 | Aza treatment increases immunogenicity

(**a**) Immunogenicity estimates in cell line grafted (4T1, B16, CT26 and MC38) mouse tumour models. Shown are TMB expressed as the number of mutations per Mb of expressed coding DNA sequence (CDS), mRNA expression (transcripts per million, TPM) of *Pdl1* and *Pd1* immune checkpoint molecules and activated $CD8^+$ T-cell enrichment estimated through gene set variance analysis (GSVA) of immune metagenes. (**b**) Growth of tumours generated by grafting mice subcutaneously with CT26 and MC38, or orthotopically with 4T1 cells. Mice were treated with anti-PD1 or control IgG antibody (see Methods). Data represent estimated mean and s.e.m. from 3 independent experiments, each with at least n=6 per group. Arrows indicate treatment times. * P<0.05 by repeated measurement analysis. (**c**) Cryptic transcript expression (average counts per million + s.e.m.) in cell line grafted (4T1, B16, CT26 and MC38) mouse tumour models. (**d**) Heatmap (*top*) and violin plots (*bottom*) showing the expression of cryptic transcripts (bound by HIF1β in 4T1 cells) in 4T1 cells WT (*Hif1b*-WT) or deficient (*Hif1b*-KO) for *Hif1b* exposed to hypoxia for 24 hours (0.5% O_2) and treated with vehicle or aza; expression of retrotransposon subfamilies using RepEnrich (*top violin plot*) and of cryptic transcripts was determined using CREDENToR (*bottom violin plot*) (at least n=5 per treatment condition). Difference in the distribution of cryptic transcript expression is denoted as fold change of counts per million over vehicle-treated and normoxic 4T1 cells. ***P<0.001 by paired t-test. (**e**) Heatmap showing the expression of a proliferation gene signature[1] in 4T1 tumours from mice treated with either aza or PBS (at least 6 mice per treatment condition were sequenced). Samples and genes were unsupervisedly clustered. Aza-treated tumours do not appear to form a more proliferative subset of samples. (**f**) Quantification of $CD8^+$ and granzyme b $(Gzmb)^+$ cells, depicted as percentages of $CD45^+$ cells and $CD8^+$ cells, from vehicle- or aza-treated 4T1 tumours bearing mice and representative immunofluorescence images (scale 50 µm). **P<0.01 by t-test. (**g**) Immunoblot analysis (*left*) and mRNA expression (log_2 fold change *versus* WT) (*right*) of HIF1β in polyclonal 4T1 cells that are WT (*Hif1b*-WT) or KO (*Hif1b*-KO) for *Hif1b* (see Methods). (**h**) Violin plot showing expression of cryptic transcripts unbound by HIF1β in 4T1 cells. Shown is expression as determined using CREDENToR in 4T1 cells WT (*Hif1b*-WT) or deficient (*Hif1b*-KO) for *Hif1b* exposed to hypoxia for 24 hours (0.5% O_2) and treated with vehicle or aza. (**i**) Expression of cryptic transcripts significantly downregulated *in vitro* (1% FDR) in hypoxic *Hif1b*-KO 4T1 cells *versus* hypoxic WT 4T1 cells, and stratified into being HIF-bound and non-HIF-bound. Expression changes are shown for 4T1 cells grown *in vitro* (*left panel*), and for 4T1 tumours orthotopically grafted in mice (*right panel*, *in vivo*). ***P<0.001; **P<0.01; *P<0.05 by t-test.

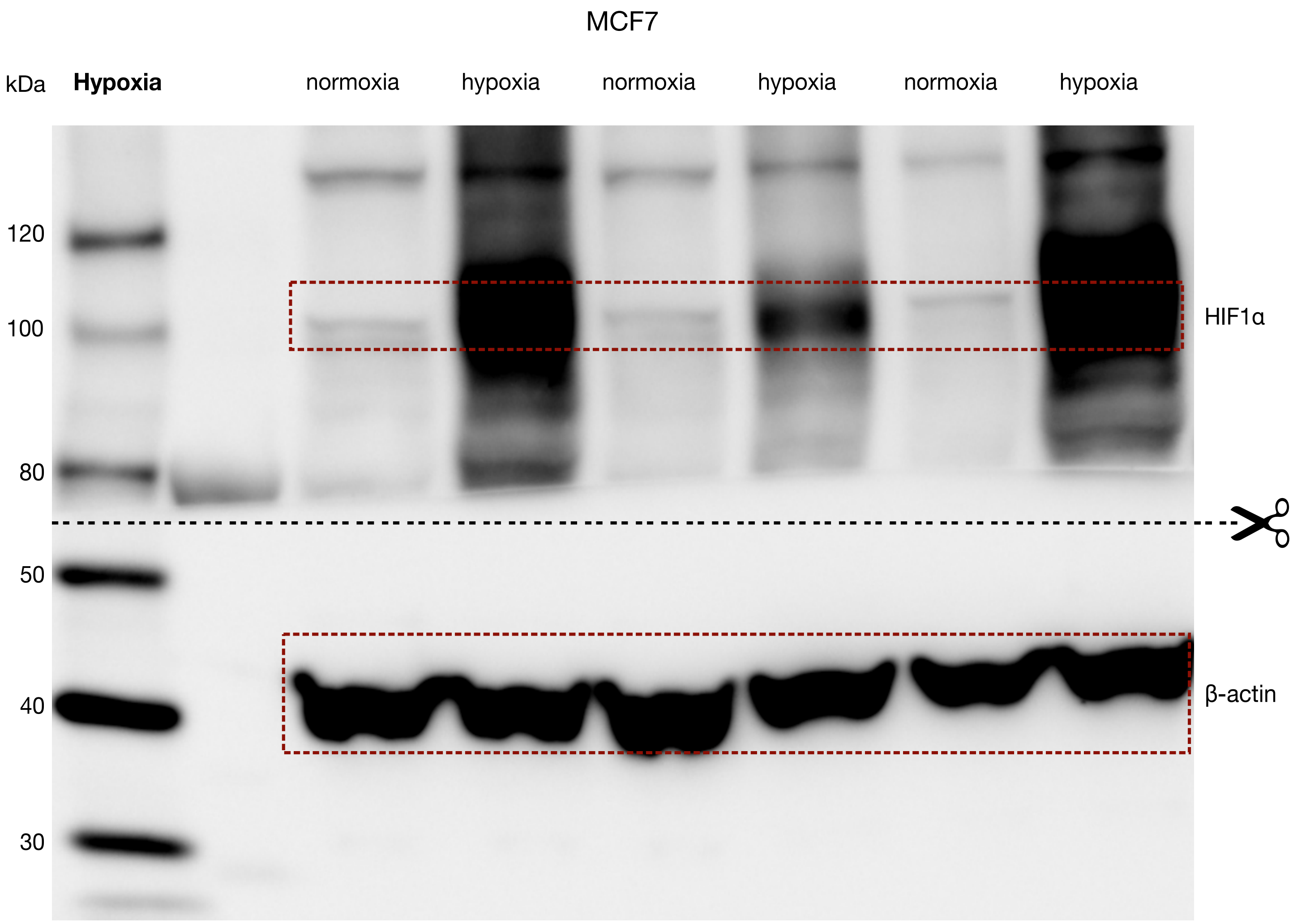
MCF7
kDa
Hypoxia
normoxia
hypoxia
normoxia
hypoxia
normoxia
hypoxia
120
100
80
50
40
30
HIF1α
β-actin

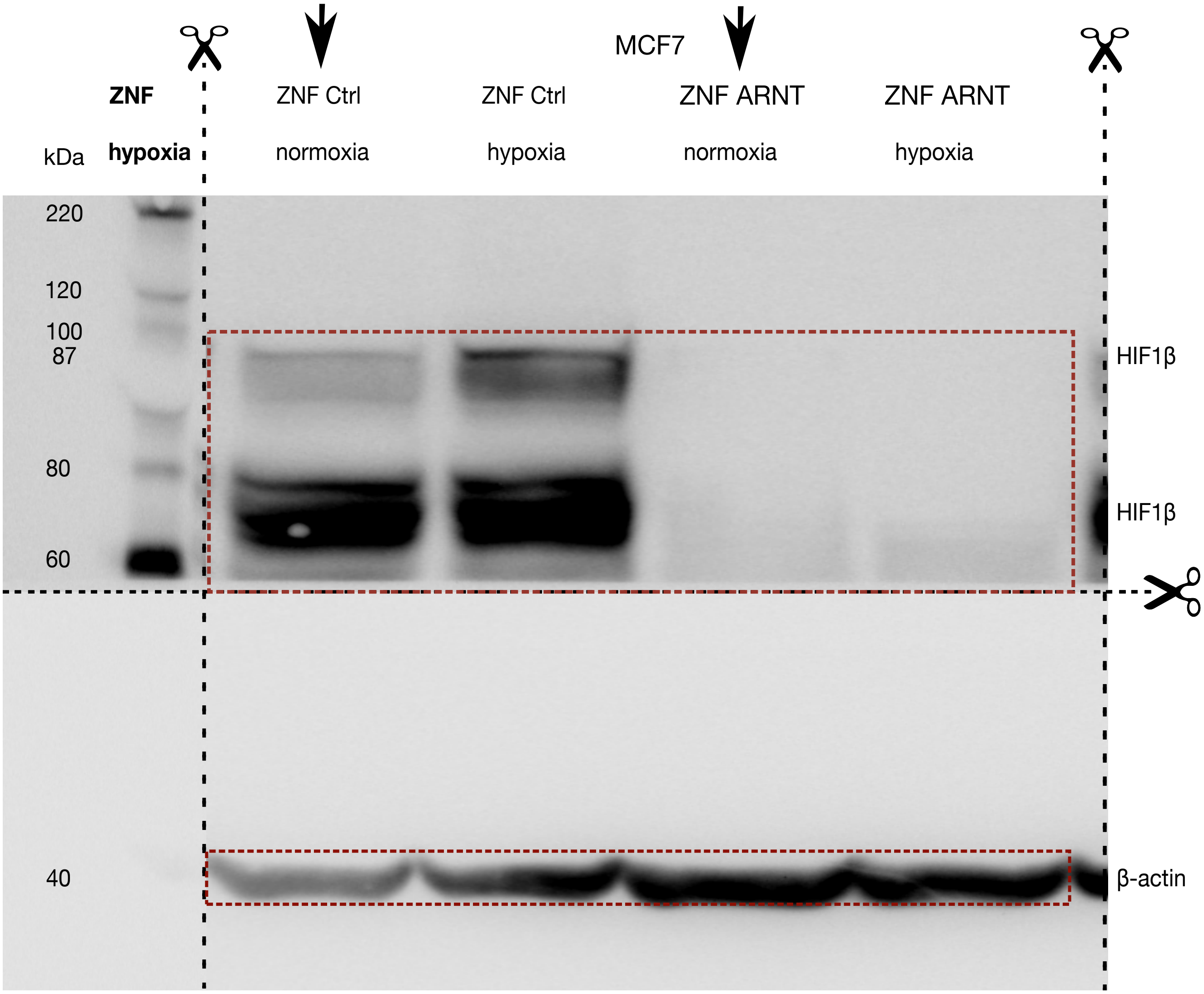
MCF7
ZNF
hypoxia
ZNF Ctrl
normoxia
ZNF Ctrl
hypoxia
ZNF ARNT
normoxia
ZNF ARNT
hypoxia
kDa
220
120
100
87
80
60
40
HIF1β
HIF1β
β-actin

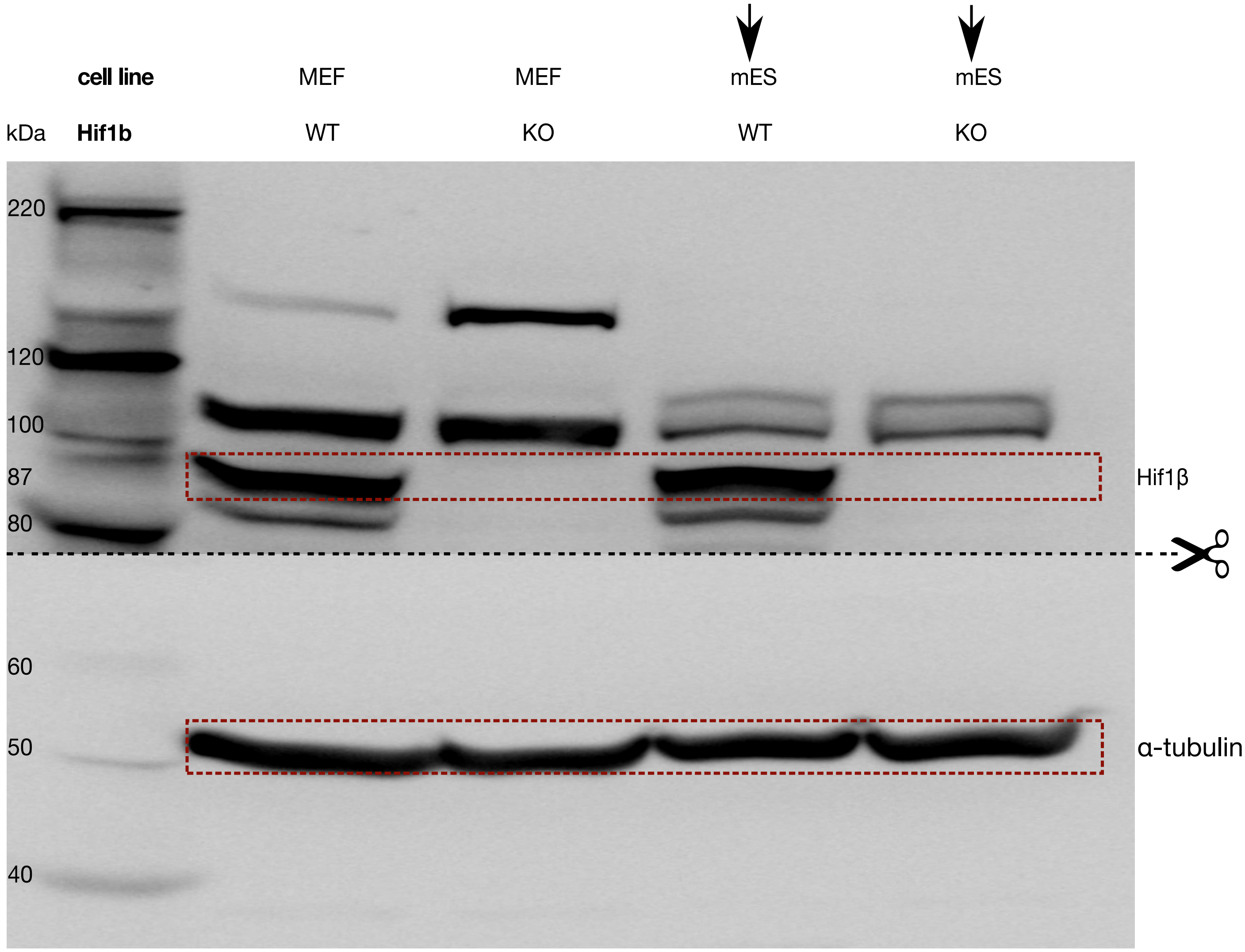
cell line
MEF
MEF
mES
mES
kDa
Hif1b
WT
KO
WT
KO
220
120
100
87
80
60
50
40
Hif1β
α-tubulin
